# Comparative analysis of deep learning and tree-based models in power demand prediction: Accuracy, interpretability, and computational efficiency

**DOI:** 10.1177/17442591251333144

**Published:** 2025-06-10

**Authors:** Bowen Yang, Mustafa Gül, Yuxiang Chen

**Affiliations:** Department of Civil and Environmental Engineering, University of Alberta, Edmonton, AB, Canada

**Keywords:** Building power prediction, accuracy, interpretability, computational efficiency, machine learning, deep learning

## Abstract

Research and development have demonstrated that effective building energy prediction is significant for enhancing energy efficiency and ensuring grid reliability. Many machine learning (ML) models, particularly deep learning (DL) approaches, are widely used for power or peak demand forecasting. However, evaluating prediction models solely based on accuracy is insufficient, as complex models often suffer from low interpretability and high computational costs, making them difficult to implement in real-world applications. This study proposes a multi-perspective evaluation analysis that includes prediction accuracy (both overall and at different power levels), interpretability (global/local perspectives and model structure), and computational efficiency. Three popular DL models—recurrent neural network, gated recurrent unit, long short-term memory, and three tree-based models—random forecast, extreme gradient boosting, and light gradient boosting machine—are analyzed due to their popularity and high prediction accuracy in the field of power demand prediction. The comparison reveals the following: (1) The best-performing prediction model changes under different power demand levels. In scenarios with lower power usage patterns, tree-based models achieve an average CV-RMSE of 13.62%, which is comparable to the 12.17% average CV-RMSE of DL models. (2) Global and local interpretations indicate that past power use and time-related features are the most important. Tree-based models excel at identifying which specific lagged features are more significant. (3) The DL model behavior can be interpreted by visualizing the hidden state at each layer to reveal how the model captures temporal dynamics across different time steps. However, tree-based models are more intuitive to interpret using straightforward decision rules and structures. This study provides guidance for applying ML algorithms to load forecasting, offering multiple perspectives on model selection trade-offs.

## Introduction

### Background

Buildings contribute significantly to global energy consumption, accounting for approximately 30% of total final energy consumption and nearly 40% of energy-related carbon dioxide emissions (Natural Resources Canada, 2024). With the global population projected to rise by around two billion by 2050 (Hagmann, 2001), building energy demand is expected to increase, posing a grid transmission capacity challenge and leading to an increase in primary energy consumption. In Canada, for example, buildings account for about 25% of total energy use, with 81% and 60% of this energy consumed for space and water heating in residential and commercial buildings, respectively. Building energy management involves systematic monitoring and control systems of energy usage in buildings (Hagmann, 2001). Accurate energy prediction models are critical to help optimize system operations based on projected consumption, thereby matching renewable energy supply and reducing peak demand (Chen et al., 2022).

Building energy modeling and prediction contains three primary approaches: “white-box” (physical model), “gray-box” (hybrid model), and “black-box” (machine learning (ML) models; Sun et al., 2020). Physical models, based on heat balance equations and physics principles, are ideal for scenarios with well-understood physical processes (Chen et al., 2022). EnergyPlus, TRNSYS, Modelica, DOE-2, eQUEST, and other commercial software have been developed for energy modeling during the building design and operation phases. However, in order to achieve accurate energy simulation, this approach relies heavily on detailed data about building characteristics, energy systems, and surrounding environmental weather conditions, which can be difficult to obtain (Li and Wen, 2014). The hybrid models, which combine physical laws with data-driven techniques, demonstrate improved prediction accuracy over traditional physical models. The resistance and capacitance network (RC) model is a typical gray model for predicting heating and cooling demand. Dong et al. (2016) developed an RC model for 1- and 24-h ahead load forecasting, and the result showed an improvement in prediction accuracy compared to other data-driven models. However, the RC model often struggles with the uncertainty of model structure (i.e. thermal network). This issue is evident in a 6R4C model, which can represent a building’s thermal behavior more accurately than a simplified 2R1C model (Li and Wen, 2014). Yet, it requires significantly detailed information about the building’s properties, which increases the model’s complexity, requiring more parameters and processing computational resources. Furthermore, RC models do not inherently consider the energy consumption of appliances, lighting, and other non-heating, ventilation, and air conditioning (HVAC) electrical loads. For total energy demand prediction, additional models need to be developed and integrated with the RC model, making the prediction model more complex to develop (Sun et al., 2020). ML models, on the other hand, do not require the construction of thermal equations. They can recognize the complex patterns and relationships between the output and the input from the data, which is very useful for dealing with non-linear systems in real-world applications (i.e. total load forecasting).

### Related work

Traditional “black-box” approaches in building energy prediction, such as support vector machine (SVM; Dong et al., 2005), *k*-nearest neighbor (KNN; Khan et al., 2019), decision tree (DT), autoregressive integrated moving average (ARIMA), and artificial neural network (ANN), have gained extensive attention due to their well-established mathematical foundations. Dong et al. (2005) used SVM to predict monthly building energy consumption in a tropical region using a 3-year dataset, demonstrating SVM’s effective performance with minimal parameter tuning. Similarly, Yu et al. (2010) employed the DT, which is known for its usability and transparent decision-making structure, to classify energy use intensity for a residential building in Japan. The authors concluded that the DT yielded a good performance result. Newsham and Birt (2010) adapted the ARIMA model, which is well-known for time-series forecasting, to predict electricity demand while taking occupancy data into account. However, it is ineffective in dealing with non-stationary problems such as residential building load forecasting (Chen et al., 2017). The ANN has become increasingly popular due to its ability to learn and model complex relationships. Xu et al. (2019) built an ANN model to forecast the load of an educational building one day in advance and demonstrated that the ANN could achieve acceptable accuracy with a mean absolute percentage error (MAPE) of 5%.

Tree-based ensemble models, including random forest (RF), extreme gradient boosting (XGBoost), light gradient boosting machine (LightGBM), and gradient-boosted decision trees (GBDT), have become prominent in recent years (Yang et al., 2021). These models are highly effective in capturing intricate input-output relationships and determining the significance of different features. Wang et al. (2018) applied RF to predict the hourly energy consumption for two educational buildings using monthly and yearly data. The authors proved that the RF is capable of predicting energy demand accurately, and the variable importance analysis can also assist building operators in understanding and managing energy usage by determining which variables are important. The study by Feng et al. (2021) developed an XGBoost model to predict the space cooling load. The results emphasized its efficiency, demonstrating superior accuracy and computational cost performance compared to eight other ML models. LightGBM is identified as a more efficient ensemble tree-based model than other existing ensemble learning models (Cui et al., 2021; Nayak et al., 2021). Guo et al. (2023) used RF to rank the essential features and determine which inputs can be fed to the LightGBM model to improve the prediction accuracy of the heating and cooling load.

Conventional ML methodologies exhibit limitations in capturing the non-linear dynamics characteristic of building operations (Li et al., 2021). As a result, there has been a shift toward adopting advanced data-driven techniques, specifically deep learning (DL) models, in recent years (Esrafilian-Najafabadi and Haghighat, 2022; Jin et al., 2019). Liu et al. (2023) implemented a gated recurrent unit (GRU) model to forecast how much energy an office building’s HVAC system would consume. They discovered that the DL model did better (*R* squared (*R*^2^) of 0.85) than the SVM and RF models. Gao et al. (2020) built a deep transfer learning model using a convolutional neural network (CNN) with an attention layer and sequence-to-sequence methods, and the model worked well even with little data. Moreover, Li et al. (2022) applied a long short-term memory (LSTM) model to predict building energy consumption over various prediction horizons (3, 6, 12, and 24 h ahead), concluding that prediction accuracy decreases as the horizon gets longer. The DL model can also be used for feature extraction. In Sekhar and Dahiya (2023), the LSTM model was combined with grey wolf optimization (GWO) and CNN to make a GWO-CNN-BiLSTM hybrid strategy. CNN was used as a one-dimensional layer for extracting spatial time-series data.

Once the models are developed, performance evaluation is essential to guarantee their reliability and suitability for practical applications. Prediction accuracy, interpretability, and computational efficiency are three critical evaluation perspectives for physical, hybrid, and ML models in energy demand prediction. Prediction accuracy is the primary metric for evaluating a model’s performance throughout the training period. It measures how closely the model’s predictions match the actual values. The metrics frequently used are mean absolute error (MAE), MAPE, root mean square error (RMSE), normalized mean bias error (NMBE), coefficient of variation of the root mean square error (CV-RMSE), mean bias error (MBE), mean square error (MSE), and *R*^2^. The nature of the work determines the criteria used. Please see Sun et al. (2020) for more explanations and practical uses of these criteria.

Interpretability refers to the degree to which a human can understand the reasons behind a model’s decision. It is becoming increasingly important in fields that require clear interpretation, such as healthcare (Vellido, 2020), finance (Florez-Lopez and Ramon-Jeronimo, 2015), self-driving cars (Kim and Canny, 2017), building energy management (Gao and Ruan, 2021), building fault detection (Gao et al., 2023), and building retrofit (Alrobaie and Krarti, 2023). The rapid progress in computer technology and the abundance of data and sensors available on the internet have accelerated the development of artificial intelligence (AI) technologies. Applications based on DL algorithms, such as generative pre-trained transformers (GPT) and AlphaGo, have gained prevalence due to their remarkable ability to accomplish tasks like humans. However, their sophisticated structures (more layers and neurons, as well as a complex model architecture) make it challenging to explain why they make certain decisions or predictions (Samek et al., 2017). Importantly, people are less likely to use a model if it lacks interpretability (Ribeiro et al., 2016b).

There are two types of explainable AI for power demand prediction: ante hoc and post hoc, and the difference is whether the models can provide explanations while being trained. Ante hoc methods use models that are naturally easy to understand, such as tree-based models (e.g. DT, RF, XGBoost, and LightGBM), rule-based models (e.g. association rule learning; Setnes et al., 1998), linear models (e.g. linear and logistic regression), and attention mechanisms. Conversely, post hoc approaches are used after model training to explain models that are not inherently interpretable. Common post hoc approaches include shapley additive explanations (SHAP), local interpretable model-agnostic explanations (LIME; Ribeiro et al., 2016a), partial dependence plots (PDP; Greenwell, 2017), and causal inference (Moraffah et al., 2020). For “ante hoc” interpretation, Zhang et al. (Zhang et al., 2021) developed an explainable DT-based surrogated model that displays the tree structures, which helps users better understand which features are important for predicting hourly energy use. Gao and Ruan (2021) proposed explainable encoder-decoder models using attention mechanisms based on LSTM to predict building energy consumption. Li et al. (2021) investigated how attention mechanisms can improve the interpretability of recurrent neural network (RNN) models to forecast the cooling demand 24 h ahead. For post hoc interpretation, Aras and Van (2022) employed the SHAP technique to explore the correlation between the inputs and output in estimating annual building energy consumption. Jin et al. (2022) developed RF and adaptive boosting trees to predict the energy usage intensity and applied LIME to determine the importance of various features for the two models. More extensive applications of explainable models in building energy management and power systems can be found in Machlev et al. (2022) and Chen et al. (2023).

Another essential metric for evaluation is computational efficiency, which relates to the computational resources, such as time and memory, required for model training and validation. The number of inputs is a critical factor that influences computational time. Chari and Christodoulou (2017) studied how varied inputs affect the training time required for an ANN model and found that adding features results in longer training times. The number of hidden layers is also a crucial indicator for DL models. The study by Singaravel et al. (Singaravel et al., 2018) examined how the number of hidden units and training parameters in LSTM affect the computational efficiency, and they found that models with more hidden layers need more time to train.

Previous studies compared the performance of different ML models, primarily focusing on aspects such as analyzing the accuracy across different prediction horizons (Dong et al., 2016; Fan et al., 2014; Runge and Saloux, 2023), investigating the impacts of data volume (Amasyali and El-Gohary, 2021; Olu-Ajayi et al., 2022), the influence of previous lag values (Liu et al., 2023), and feature engineering on accuracy (Ahmad et al., 2017; Esrafilian-Najafabadi and Haghighat, 2022; Zhou et al., 2021). As shown in Table 1, many authors generally evaluate ML models based on one or two criteria, neglecting a comprehensive assessment. Notably, none of these studies evaluate accuracy at different power/energy levels or provide model structure interpretation. However, focusing on only one or two evaluation aspects is insufficient for a holistic view of model performance. Consequently, a critical gap remains in the systematic performance comparison between tree-based and DL models across multiple evaluation dimensions.

**Table 1.** A summary of the performance comparisons of various ML models from different evaluation criteria.

Studies	Buildingtype	Models	Multi-perspective evaluation analysis				
			Overall accuracy	Accuracyat differentpower/energylevels	Prediction interpretation	Modelstructureinterpretation	Computationalefficiency
Ahmad et al. (2017)	Commercial	ANN/RF	✓	✗	✓	✗	✗
Wang et al. (2019)	Residential	ANN/SVM/RF/ GBDT/XGBoost	✓	✗	✓	✗	✓
Wang et al. (2020)	Residential	MLR/RF/ANN/ RNN	✓	✗	✗	✗	✗
Liu et al. (2019)	Residential/ Commercial/ Educational	ANN/SVM	✓	✗	✗	✗	✓
Seyedzadeh et al.(2019)	Residential/ Commercial	ANN/SVM/GP/ RF/GBDT	✓	✗	✓	✗	✓
Fan et al. (2019b)	Educational	GLM/ANN/SVM/ RF/XGBoost	✓	✗	✓	✗	✗
Chakraborty and Elzarka (2019)	Commercial	XGBoost/ANN/OLS	✓	✗	✗	✗	✗
Pham et al. (2020)	Commercial	RF/M5 tree/RT	✓	✗	✗	✗	✗
Wu et al. (2024)	Residential/ Commercial/ Industrial/ Educational	MLR/RR/ LassoR/ BayesianRR/SVM/ ANN/GBDT/ XGBoost	✓	✗	✓	✗	✗
Zhou et al. (2021)	Educational/ Commercial	ANN/RBFNN/ELM/GRNN/SVM/GPR/ LS-SVM/RTree/ M5 tree/RF/MARS/ GBDT/XGBoost/ LightGBM/ CatBoost	✓	✗	✗	✗	✓
Deng et al. (2018)	Commercial	LR/LassoR/SVM/ GBDT/ANN	✓	✗	✗	✗	✗
Ahmad et al. (2018)	Commercial/ Industrial	DT/GP/stepwise LR/generalized LR	✓	✗	✗	✗	✗
Olu-Ajayi et al. (2022)	Residential	GBDT/RF/KNN/ SVM/DT/LR/DNN	✓	✗	✗	✗	✗
Walker et al. (2020)	Commercial	Boosted tree/RF/SVM/ ANN	✓	✗	✗	✗	✗
Chalapathy et al. (2021)	Commercial	RNN/RNN-MIMO/ S2S	✓	✗	✗	✗	✓
This study	Residential	RF/XGBoost/ LightGBM/ RNN/GRU/LSTM	✓	✓	✓	✓	✓

MLR: Multiple linear regression; GP: Gaussian process; GLM: generalized linear models; OLS: ordinary least square; RT: random tree; RR: ridge regression; LassoR: lasso regression; RBFNN: radials basis function neural network; GRNN: generalized regression neural network; ELM: extreme learning machine; LS-SVM: least-square SVM; GPR: Gaussian process regression; RTree: regression tree; MARS: multi-adaptive regression spline; CatBoost: category boosting tree; DNN: deep neural network; MIMO: multiple input multiple output; S2S: sequence to sequence.

### Innovation of this study

This study aims to bridge this gap by systematically comparing and analyzing the performance of DL and tree-based models, considering not only their predictive capabilities but also their interpretability and computational efficiency. The key innovations of this study are:

- Unlike many previous comparison analysis studies that focus on classical ML models, this study conducts a systematic performance comparison specifically between tree-based and DL models, providing a comprehensive understanding of their capabilities and limitations.
- This study goes beyond overall prediction accuracy by evaluating accuracy at different power levels. This approach offers insights into model performance under various power consumption scenarios, which is crucial for practical applications.
- This study uniquely provides insights into the internal workings of DL models through visualizations of hidden states and compares their model structure interpretability with tree-based models. This detailed comparison enhances understanding of the “black-box” nature of DL models and the relative interpretability of tree-based models, underscoring the importance of model transparency.

## Methodology

**Figure 1.**
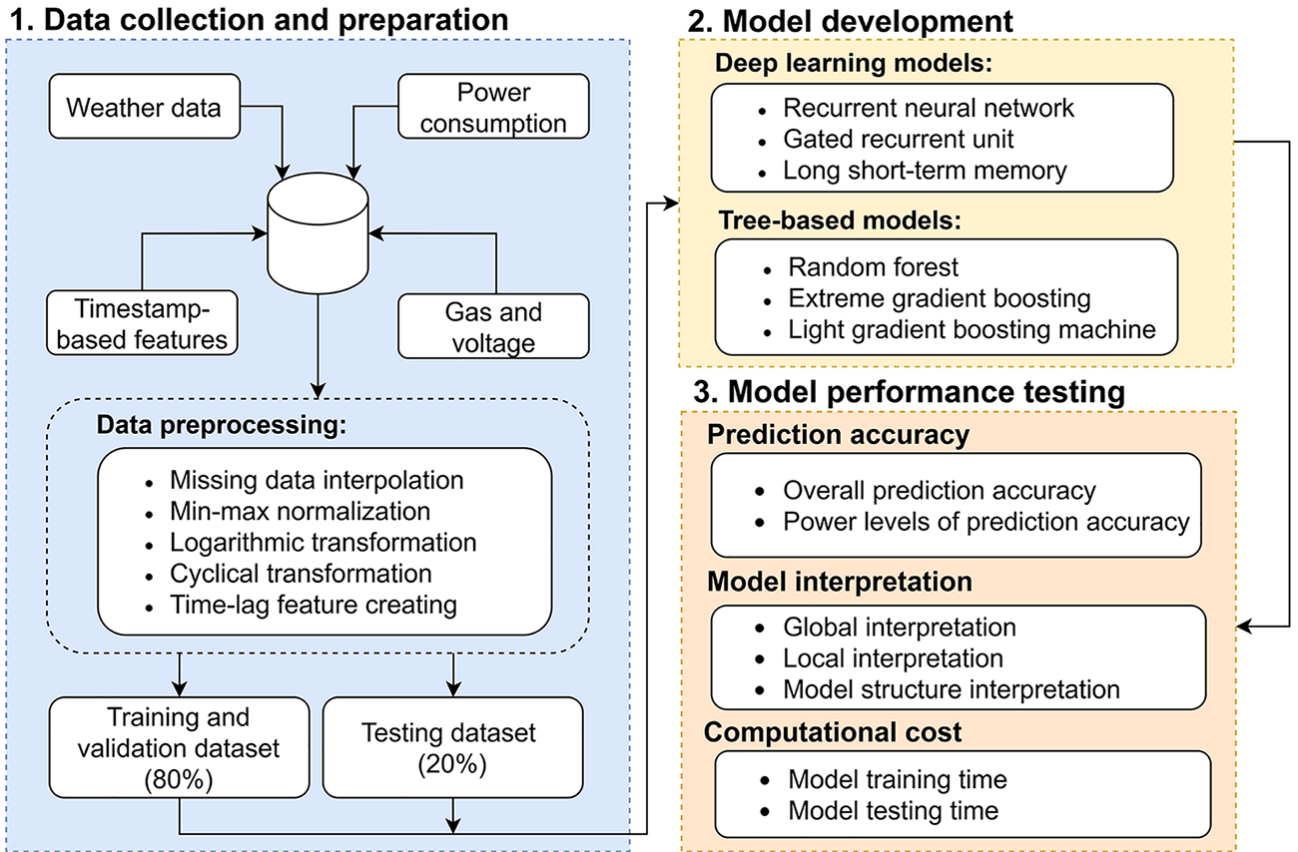
Methodology overall flow.

This section presents the methodology overall flow, machine learning model descriptions, and the methods used to evaluate prediction accuracy, model interpretability, and computational efficiency. These steps provide a systematic framework for developing and assessing the models. The overall workflow used in this study is shown in Figure 1. Below is a brief overview:

***Data collection and preparation***: The data was collected in two residential units with a 1-h time interval. Details of the dataset are provided in Section 2.1. To ensure data quality, preprocessing steps included missing data interpolation, min-max normalization, logarithmic transformation, and cyclical transformation techniques.
***Model development***: Three deep learning and three tree-based models were used in this study. Their descriptions and configurations are detailed in Section 2.2.
***Model performance testing***: The models were evaluated from three perspectives. Further details are provided in Sections 2.3, 2.4, and 2.5.

### Case study and data preparation

**Figure 2.**
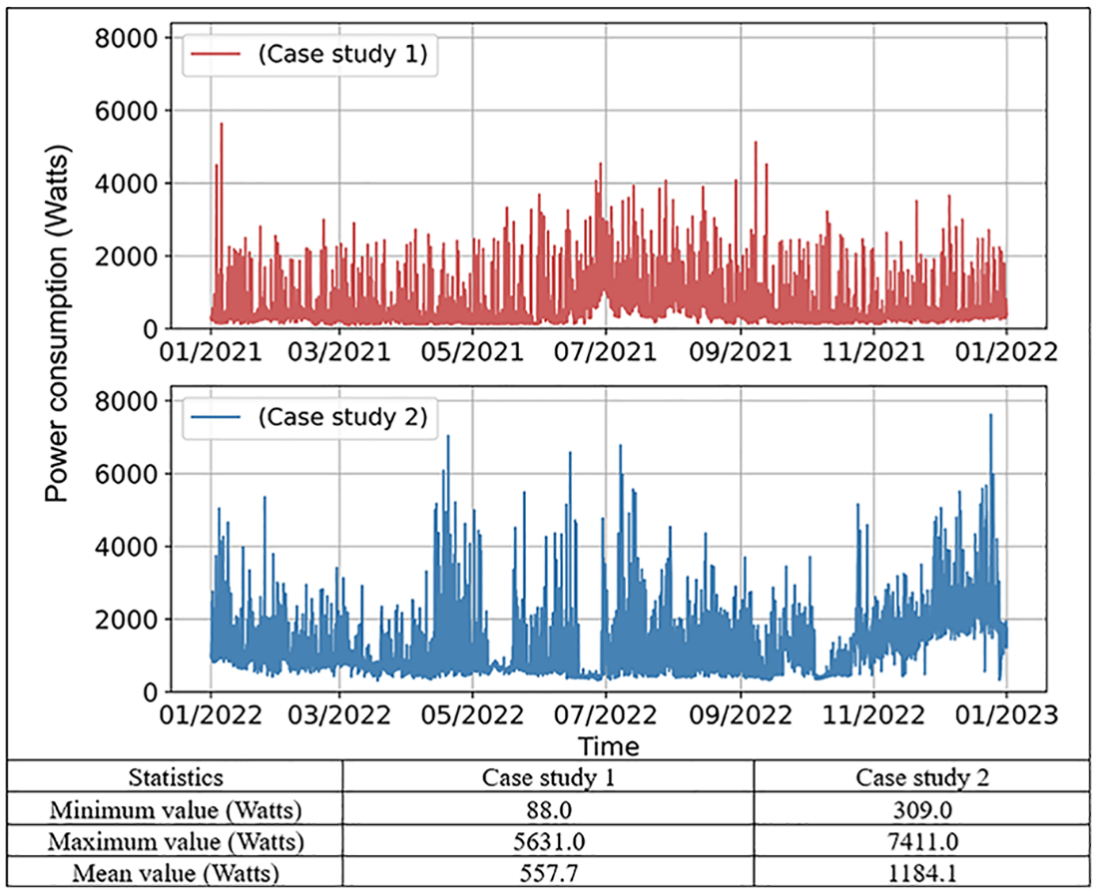
Hourly power consumption with statistics of two case studies.

To train and verify the performance of different ML models, measured data was retrieved from two units in multi-unit residential communities in Edmonton, Canada, which are representative of typical building types in the region. Case study 1 involves a townhouse with an area of 135 m^2^, and case study 2 involves a duplex household with an area of 353 m^2^. To maximize energy use for space heating, each unit is equipped with an efficient hybrid heating system that consists of an electric air-source heat pump and a natural gas tankless water heater to optimize energy use. Figure 2 presents the power consumption data for the two case studies over a year. Case study 1 shows a lower average consumption, whereas case study 2 has a higher average consumption. This difference is due to several factors, such as building size (the area of the house in case study 2 is 2.6 times that of case study 1), usage patterns, and the type of equipment used. The accompanying table provides a summary of the power consumption characteristics for both case studies, highlighting the significant disparities in minimum, maximum, and average power consumption values. Hourly data from a full year was collected and analyzed for this study. The dataset spans the period from January 1, 2021, to December 31, 2021, and January 1, 2022, to December 31, 2022, for case studies 1 and 2, respectively, to provide a comprehensive view of the building’s energy consumption patterns throughout different seasons at different units. The data can be classified into weather conditions, time-related features, previous power consumption, and others. Weather data were gathered from the Edmonton weather station, which captured the environmental data. Power consumption data were collected using a circuit-level monitoring system. Time-index characteristics, such as the month, day, and hour, were created and converted into cyclical features to reflect periodic patterns. Table 2 displays the details of the input variables, which were used to train the models, and the target variable was the total power demand in the next hour. The dataset was divided into 64% for training (2021/2022 Jan. 1st, 0:00–2021/2022 Aug. 22nd, 13:00), 16% for validation (2021/2022 Aug. 22nd, 14:00–2021/2022 Oct. 19th, 22:00), and 20% for testing (2021/2022 Oct. 19th, 23:00–2021/2022 Dec. 31st, 23:00). This division was chosen based on the “rule of thumb” and practices observed in prior studies. It is a common approach to allocate approximately 60%–70% of the data for training and the rest for validation and testing. It is worth mentioning that this study focuses on a detailed comparison of case study 1, while the purpose of case study 2 is to make a comparison with case study 1, with further details available in the Appendix B, C, and D.

**Table 2.** Input variables.

Variable category	Input	Type	Unit
Weather condition	Outdoor temperature	Numeric	°C
	Dewpoint temperature	Numeric	°C
	Relative humidity	Numeric	%
	Wind speed	Numeric	km/h
	Atmospheric pressure	Numeric	kPa
Time-index features	Month of the year	Categorical	January–December
	Day of the week	Categorical	Monday–Sunday
	Time of the day	Categorical	0:00–23:00
Power consumption	Total electric powerconsumption/electrical power consumption ofheat pump, air handling unit, and heat recoveryventilator	Numeric	Watts
Other	Gas consumption	Numeric	Cubit foot
	Voltage	Numeric	Volt

### Model description

This study covers six ML algorithms and the selection of these models was motivated by their widespread use and effectiveness in building power demand prediction. DL models, particularly RNN, GRU, and LSTM, have demonstrated strong performance in capturing temporal dependencies in building load forecasting (Fan et al., 2019a). On the other hand, tree-based models such as RF, XGBoost, and LightGBM are known for their robustness in handling non-linear relationships and have been widely applied in energy prediction tasks (Liu et al., 2022). Notably, tree-based models were demonstrated as the most popular and accurate ML methods in the ASHRAE Great Energy Predictor III competition, showing their effectiveness in power demand prediction (Miller et al., 2020). The parameter settings for the models are shown in Table 3. The hyperparameters were determined considering multiple factors. Initially, many hyperparameters were selected based on widely accepted best practices for the algorithms, which were established in previous studies. Then, different configurations were tested through multiple trials to identify the settings that have the best performance. During this process, hyperparameters were fine-tuned by evaluating the trade-offs between model accuracy and computational efficiency. More details of ML models’ principles can be found in Appendix A.

**Table 3.** Hyperparameters settings of different models.

Algorithm	Hyperparameter	Value
RNN	Layers	2
	Units	(64, 64)
	Dropout rate	0.3
	Learning rate	0.0005
	Activation function	ReLu
GRU	Layers	2
	Units	(32, 64)
	Dropout rate	0.3
	Learning rate	0.0005
	Activation function	ReLu
LSTM	Layers	2
	Units	(64, 64)
	Dropout rate	0.3
	Learning rate	0.0001
	Activation function	ReLu
RF	Number of trees	100
	Maximum depth of the tree	20
	Minimum number for internal node subdivision	12
XGBoost	Number of trees	42
	Maximum tree depth	5
	Learning rate	0.1
LightGBM	Number of trees	300
	Maximum depth of the tree	8

#### Recurrent neural network model

The RNN is a type of neural network designed specifically to process sequential data. Unlike traditional feedforward neural networks, which propagate information unidirectionally from input to output layers without feedback loops (Pang et al., 2020), RNN has a recurrent connection that allows them to retain a form of “memory” of previous inputs. This feature enables the network to exhibit dynamic temporal behavior.

#### Gated recurrent unit model

The GRU is a variant of the RNN architecture optimized for handling sequential data with more complex internal mechanisms. The GRU improves upon the basic RNN by incorporating two gates: the update gate and the reset gate, which allow the network to capture dependencies at different time scales more effectively.

#### Long short-term memory model

The LSTM, introduced by Schmidhuber and Hochreiter (1997), represents an advanced RNN architecture designed to learn long-term dependencies in sequence prediction problems. The “long” and “short” in LSTM refer to the types of memory that the model aims to keep. Short-term memory refers to the model remembering information from a few time steps earlier, and long-term memory refers to the model preserving information, taking many steps back in the sequence. The LSTM network includes a three-gate mechanism: the forget gate, input gate, and output gate, which adjusts the flow of information within the cell. This structure enables the LSTM to disregard less useful historical information while retaining important information over longer periods (Chung et al., 2014; Yu et al., 2019), which is beneficial for predicting future building energy consumption.

#### Random forest model

RF is a robust ensemble learning method that generates many decision trees during training. The principle of an RF is that a group of “weaker learners (i.e. individual DT)” collaborate to create a “stronger learner” to make predictions. RF uses information gain and entropy to split the nodes. Scikit-learn uses MSE by default, which is the average of the squares of errors between the estimated and actual values.

#### Extreme gradient boosting model

Different from the RF, XGBoost is a gradient-boosting algorithm that builds each tree sequentially and combines each “weak learner (decision tree)” to achieve a “stronger learner.” Each tree is constructed based on the residual of all observations, attempts to correct the errors made by the previous tree, and implements a level-wise growth strategy to grow the level of the trees by level, which means that all nodes at a given level will split to form a balanced tree.

#### Light gradient boosting machine model

LightGBM is another gradient-boosting method developed by Microsoft in 2017 (Ke et al., 2017), which has a similar process to the XGBoost model. LightGBM employs a leaf-wise (vertical) tree growth strategy instead of traditional leaf level-wise (horizontal). This innovative technique involves selecting the leaf with the maximum loss to grow, considering all possible splits, and choosing the one that yields the highest gain in reducing MSE. Such a strategy allows the LightGBM to achieve higher efficiency and faster convergence.

### Prediction accuracy evaluation

The overall prediction accuracy was assessed by comparing predicted values to actual data from the testing dataset. Furthermore, different power levels were grouped to better understand the models’ performance in various power consumption scenarios, such as peak and off-peak periods. The model’s prediction accuracy is validated using five performance metrics: MAE, MAPE, RMSE, NMBE, and CV-RMSE.

MAE and RMSE are scale-dependent and should be used when the data scale is consistent and comparisons are within the same dataset. MAPE, NMBE, and CV-RMSE are unaffected by scale and can thus be used to compare model performance across datasets or scales. We can gain a holistic understanding of our models’ predictive capabilities and accuracy by employing these diverse metrics. Equations (1)–(5) show the definitions of these four metrics.

(1) $$\mathrm{MAE}=\frac{1}{n}\sum_{i=1}^{n}\left|y_{i}-{\hat{y}}_{i}\right|$$

(2) $$\mathrm{MAPE}=\frac{1}{n}\sum_{i=1}^{n}\left|\frac{y_{i}-{\hat{y}}_{i}}{y_{i}}\right|$$

(3) $$\mathrm{RMSE}=\sqrt{\frac{\sum_{i=1}^{n}{\left(y_{i}-{\hat{y}}_{i}\right)}^{2}}{n}}$$

(4) $$\mathrm{NMBE}=\frac{\frac{1}{n}\sum_{i=1}^{n}(y_{i}-{\hat{y}}_{i})}{\frac{\sum_{i=1}^{n}y_{i}}{n}}$$

(5) $$\mathrm{CV}-\mathrm{RMSE}=\frac{\sqrt{\frac{\sum_{i=1}^{n}{\left(y_{i}-{\hat{y}}_{i}\right)}^{2}}{n}}}{\frac{\sum_{i=1}^{n}y_{i}}{n}}$$

where $y_{i}$ represents the actual power consumption, ${\hat{y}}_{i}$ denotes the predicted power consumption and n is the number of observations.

### Model interpretation techniques

The inherent complexity of “black-box” models poses a significant challenge for end-users, as it hinders understanding and trust in the model’s predictions. To address this issue, model interpretation in this study is proposed based on three key perspectives: (1) *global interpretation* was used to analyze how model decisions are made by implementing techniques such as feature contribution ranking. (2) *local interpretation* focuses on individual predictions to explain a single observation. (3) *model structure explanation* focuses on understanding how the architecture and parameters contribute to the model’s decision-making process. Both perspectives directly impact how well humans understand the model behavior (Ribeiro et al., 2016a). In this study, SHAP values were used to explain the model, which was proposed by Lundberg and Lee (2017) and was inspired by cooperative game theory to quantify the contribution of each feature to the prediction outcome, thereby providing insight into the significant features. The SHAP values are calculated as follows:

(6) $$\varphi_{i}\left(f,x\right)=\sum_{z^{\prime }\subseteq x^{\prime }}\frac{\left|z^{\prime }\right|!(M-\left|z^{\prime }\right|-1)!}{M!}[f_{x}\left(z^{\prime }\right)-f_{x}(z^{\prime }\setminus i)]$$

where $\varphi_{i}$ denotes the SHAP value for feature i , f is the model, and x is the input data. For a single observation, all possible feature subsets need to be considered, which are denoted by $z^{\prime }$ , and $x^{\prime }$ are the subset of features used in the calculation. The contribution of a feature can be obtained by calculating the difference in predicted outcomes with ( $f_{x}(z^{\prime })$ ) and without $\left(f_{x}(z^{\prime }\setminus i\right)$ ) the feature. The backslash symbol in the equation refers to set subtraction, meaning to remove a specific feature from the set. Each possible combination of subsets is weighted using $\frac{\left|z^{\prime }\right|!(M-\left|z^{\prime }\right|-1)!}{M!}$ , with M representing the total number of features. Additionally, the model’s interpretation was investigated using visualization techniques. The hidden states were visualized for DL models to gain insights into the model’s internal representations. In contrast, we examined the tree structures to understand the decision paths and rules that produce predictions in tree-based models.

### Computation efficiency

Computational efficiency was measured by recording the time taken to train the models on the training dataset and the time required to perform predictions on the testing dataset. All models in this study were implemented using Python and tested on a PC equipped with an AMD Ryzen 5 PRO 1500 Quad-Core Processor (3.50 GHz) and 32 GB of RAM. Please note that all models were tested under the same operating conditions, minimizing external variables.

## Results and discussion

This section compares the prediction accuracy and computational efficiency between DL and tree-based models using two case studies. Given the fact that the decision-making structure and interpretability of tree-based models are better than DL models, model interpretation analysis is primarily applied to case study 1. The detailed results and discussion are shown below.

### Prediction accuracy comparison

#### Overall prediction accuracy comparison

Table 4 presents the results for both the validation and testing datasets for case study 1. The CV-RMSE for all models is less than 30% and the NMBE is below 10% for the testing dataset, which meets the requirement of ASHRAE Guideline 14-2014 (Haberl et al., 2005). According to this guideline, the CV-RMSE of hourly energy consumption should not exceed 30%, and the NMBE should be within 10% (Sun et al., 2018). The results demonstrate the proposed models’ capability for 1-h-ahead power demand prediction. In general, DL algorithms, particularly the GRU and LSTM models, outperform tree-based models across all metrics for both validation and testing datasets. In the testing dataset, the GRU model shows remarkable accuracy, with the lowest values in all metrics (MAE (46.98), MAPE (9.51%), RMSE (88.48), NMBE (2.17%), and CV-RMSE (20.68%)). This is due to their ability to capture complex temporal dependencies in time-series data using a gated mechanism. LightGBM, the best-performing tree-based model, closely followed the accuracy of the GRU model, demonstrating its efficiency in handling high-dimensional data and approximating non-linear relationships.

**Table 4.** Evaluation results of the accuracy metrics for the prediction models for case study 1.

Metrics	Validation					
	DL-based Models			Tree-based models		
	RNN	GRU	LSTM	RF	XGBoost	LightGBM
MAE	60.92	66.18	61.12	90.57	74.74	68.59
MAPE	12.71%	13.00%	12.00%	17.62%	14.70%	13.85%
RMSE	106.53	108.47	105.38	152.76	123.59	114.94
NMBE	−8.08%	−5.33%	−2.84	−0.66	−2.26	−1.83
CV-RMSE	23.20%	23.63%	22.97%	32.76%	26.4%	24.6%
Metrics	Testing					
	RNN	GRU	LSTM	RF	XGBoost	LightGBM
MAE	52.07	46.98	48.30	65.51	61.05	55.61
MAPE	10.14%	9.51%	9.53%	13.5%	12.91%	11.17%
RMSE	101.43	88.48	92.21	116.59	115.61	112.26
NMBE	−2.18%	2.17%	2.93%	2.05%	1.56%	2.08%
CV-RMSE	23.74%	20.68%	21.54%	26.87%	26.62%	25.83%

Applying the models to case study 2 (Appendix B: Overall prediction accuracy comparison for case study 2) reveals that DL models maintain higher accuracy than tree-based models. However, their performance advantage decreases when compared to case study 1, tree-based models achieve improved accuracy with the dataset from case study 2. Notably, both RMSE and MAE metrics indicate higher errors for all models in case study 2, attributed to larger actual value ranges within this dataset. Conversely, lower MAPE and CV-RMSE values in case study 2 imply a reduction in relative error, providing insights into the models’ error dynamics across different datasets.

**Figure 3.**
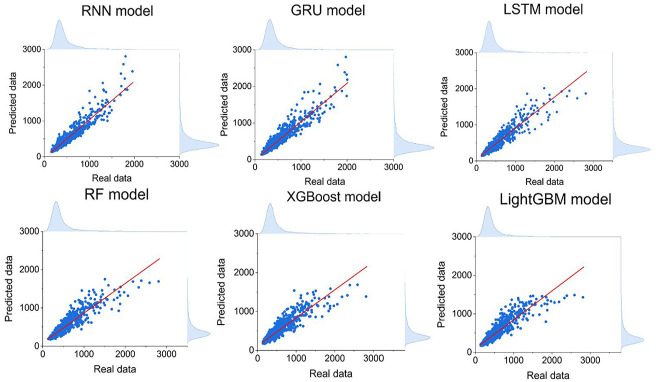
Correlation comparison of different models for case study 1 (unit: watts).

The scatter plots of predicted and actual values for all six models are shown in Figure 3. The vertical axis denotes the predicted values, and the horizontal axis represents the actual values. Overall, all models are found to cluster along the diagonal red line, indicating accurate predictions. However, deviations occurred at higher power consumption levels (around 1800 W), where models struggle with extreme values, meaning that the models cannot handle the values at the extreme peak demand. This is a common challenge in predicting energy consumption, as noted in other studies (Abdulalim Alabdullah et al., 2022; Cui et al., 2016; Liu et al., 2021; Runge and Saloux, 2023). One of the most important factors is occupant behavior, which includes tenants’ activities and preferences. Their schedules can vary and are often spontaneous, making energy usage patterns more random. For example, the energy usage of appliances, heating or cooling, and lighting systems can change dramatically due to tenants’ adjustments, which are not always predictable.

**Figure 4.**
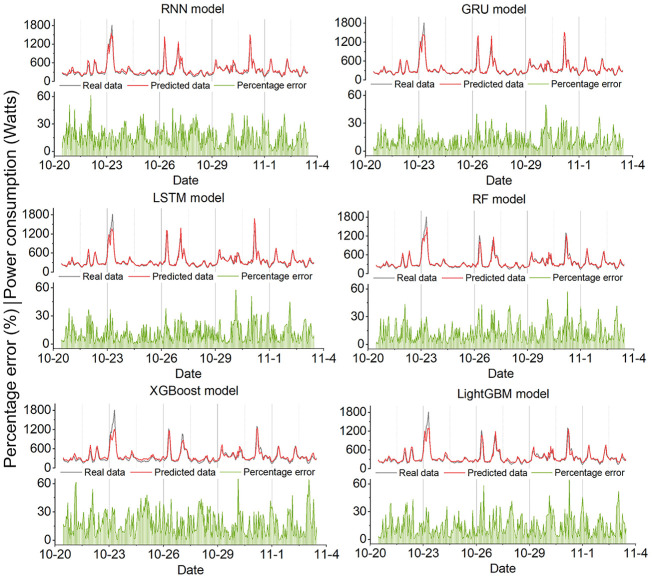
The actual and predicted values of different models for case study 1.

**Figure B1.**
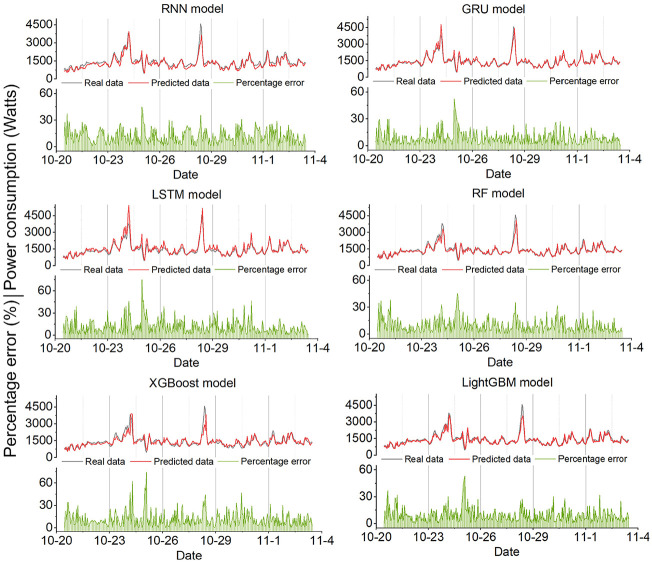
The actual and predicted values of different models of case study 2.

Figure 4 depicts the prediction results of different models for case study 1. According to Figure 4, all models demonstrate their ability to trace the trend of the actual data, indicating a solid understanding of the power consumption patterns over time. Notably, the RNN and LSTM models track fluctuations accurately, indicating their ability to capture sequential dependencies. Although the RF and XGBoost models are generally accurate, they show bigger differences when the power demand is at its highest, indicating potential difficulties in addressing non-linear interactions within the data. Increases in percentage error around the peaks show that all models struggle to predict accurately during peak power demand. These deviations may be attributed to factors that are not fully captured by the models, such as sudden changes in occupant behavior or extreme weather conditions. However, high percentage errors can also occur during periods of low power consumption. When actual power demand is low, even a small absolute prediction error can lead to a disproportionately high percentage error. This is because power consumption during low-demand periods tends to be more stochastic and influenced by minor fluctuations, making it more challenging for models to capture accurately. Figure B1 shows the prediction results of different models in case study 2. Overall, all models can capture the power demand trending, even if the peak demand data is higher than in case study 1, some models can also shorten the gap between the predicted and real data, such as GRU and RF.

**Figure 5.**
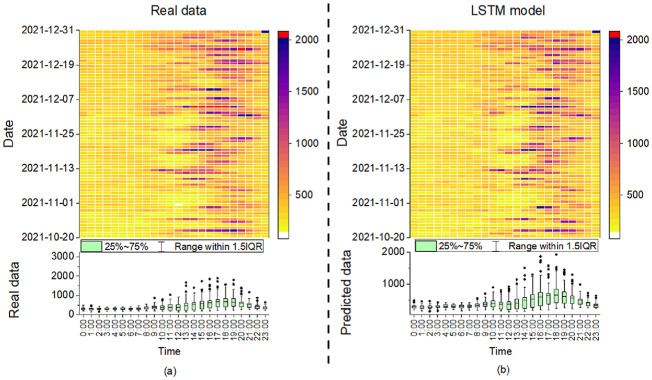
Heatmap with the building power consumption distribution: (a) real data and (b) for the predicted data made by the LSTM model. 1.5 interquartile range (IQR) represents the range of data that is within 1.5 times the IQR from the 25th and 75th percentiles.

Figure 5 offers an in-depth comparative analysis of power consumption using a heatmap to visualize intensity and a box plot to visualize the distribution of power demand throughout the day over the given period, and both are intended to provide a detailed temporal analysis of the predicted data. Since all models’ heatmaps of power usage patterns are similar, the LSTM is chosen as an example for illustration purposes. Panel (a) shows the actual power consumption, while panel (b) displays the LSTM model’s predictions. Figure 5(a) reveals the power usage pattern data of a unit over time, with darker colors representing higher power consumption values, which typically peak in the afternoon to early evening. On a specific date, take December 5th, between 14:00 and 18:00 as an example, unusually high consumption is observed, indicating that a special event was taking place during this time, resulting in increased energy demand. The same finding can also be found in Figure 5(b), but LSTM does not predict the extreme values well. More information on which features lead to predicted outcomes can be found in Section 3.2.2.

#### Prediction accuracy at different power levels

Understanding overall model prediction accuracy provides a general insight into prediction accuracy. However, it needs to illuminate how each model performs across specific power consumption levels for two reasons: first, power usage in residential buildings can vary significantly, and analyzing model behavior at different levels is essential for identifying strengths and weaknesses in specific ranges. Second, while it is common in previous studies to compare models’ overall accuracy using metrics like CV-RMSE and MAPE, these metrics do not reveal whether a model consistently outperforms others across different scenarios. By examining models’ accuracy at different power levels, we can better understand the consistency of each model’s predictions. To address this, we grouped true and predicted data into five distinct power levels, enabling a detailed analysis of model accuracy at each level. The finding reveals that no single model consistently outperforms others across all power levels. Surprisingly, despite the LSTM model showing better overall performance, it only leads at some levels. For example, a simpler RNN model demonstrates high accuracy at three different levels, thereby challenging the notion that model complexity correlates directly with enhanced performance.

In Table 5, red and green colors represent the best-performing models within each category for the corresponding power level. Specifically, red highlights the best-performing DL model, while green highlights the best-performing tree-based model. The results in Table 5 show that no single model consistently outperforms others across all power levels. This indicates that model selection should not rely solely on overall metrics but should also consider the specific characteristics of the data and the application requirements. DL models exhibit a clear advantage as power consumption increases. This variation in model accuracy is most likely due to different power usage characteristics, which are determined by the various appliances in use and the occupant behaviors. For example, some periodic appliances, such as air handling unit, heat recovery ventilator, tankless water heater, and refrigerators typically operate below 500 watts. In this range, tree-based models have similar prediction accuracy to the DL models, given the more straightforward nature of the data relationships that these models can capture with less complex decision rules. As power consumption increases, reflecting the operation of more energy-intensive devices like dryers and stoves, DL models may be more suitable for capturing the nonlinear and complex interaction between variables when dealing with high power demands.

**Table 5.** Prediction accuracy at different power levels for case study 1.

Powerlevel (Watts)	Metrics	DL-based models			Tree-based models		
		RNN	GRU	LSTM	RF	XGBoost	LightGBM
<500	MAE	32.85	27.36	27.04	30.53	36.06	30.66
	MAPE	10.60%	8.49%	8.30%	9.58%	11.86%	9.75%
	RMSE	43.19	37.88	37.87	42.31	47.93	42.86
	NMBE	−5.95%	−0.72%	0.76%	−2.15%	−4.12%	−2.41%
	CV-RMSE	13.26%	11.63%	11.63%	12.99%	14.72%	13.16%
500–1000	MAE	83.35	86.43	90.68	103.74	104.73	102.48
	MAPE	12.51%	12.50%	13.18%	15.10%	15.44%	15.03%
	RMSE	105.71	117.68	120.22	138.78	139.79	137.77
	NMBE	−1.25%	4.50%	4.26%	2.96%	3.55%	2.47%
	CV-RMSE	15.85%	17.64%	18.02%	20.81%	20.96%	20.65%
1000–1500	MAE	166.14	174.06	187.15	189.30	197.59	179.46
	MAPE	14.02%	14.76%	15.84%	15.74%	16.57%	14.97%
	RMSE	198.04	218.32	234.22	228.99	236.86	221.20
	NMBE	5.27%	4.23%	2.64%	10.98%	12.74%	11.19%
	CV-RMSE	16.51%	18.20%	19.52%	19.09%	19.74%	18.44%
1500–2000	MAE	289.66	322.49	335.68	452.98	530.15	484.68
	MAPE	16.71%	18.63%	19.35%	25.93%	30.41%	27.60%
	RMSE	376.07	407.05	408.05	502.17	577.40	540.91
	NMBE	16.84%	15.73%	16.27%	24.52%	30.62%	28.19%
	CV-RMSE	21.87%	23.67%	23.73%	29.20%	33.58%	31.46%
>2000	MAE	675.30	538.32	621.80	790.06	936.57	991.26
	MAPE	28.00%	22.14%	25.65%	32.71%	38.91%	41.07%
	RMSE	708.81	589.03	664.42	813.42	963.70	1010.84
	NMBE	28.22%	22.50%	25.99%	33.02%	39.14%	41.43%
	CV-RMSE	29.62%	24.62%	27.77%	34.00%	40.28%	42.25%

In case study 2, as shown in Appendix C: Prediction accuracy at different power level for case study 2. The GRU model among the DL models and the RF and LightGBM models among the tree-based models demonstrated high accuracy at most power levels. In both cases, the prediction errors (i.e. MAE and RMSE) tend to increase with power levels. However, CV-RMSE did not follow a consistent trend, which means that higher power levels do not necessarily lead to proportionately higher errors. In contrast to case study 1, an interesting finding was observed in case study 2: the models performed less effectively at regular power levels (below 500 watts) but performed well for higher power demand. This variability could add noise to the data and make it hard to predict.

### Model interpretation

#### Global interpretation

**Figure 6.**
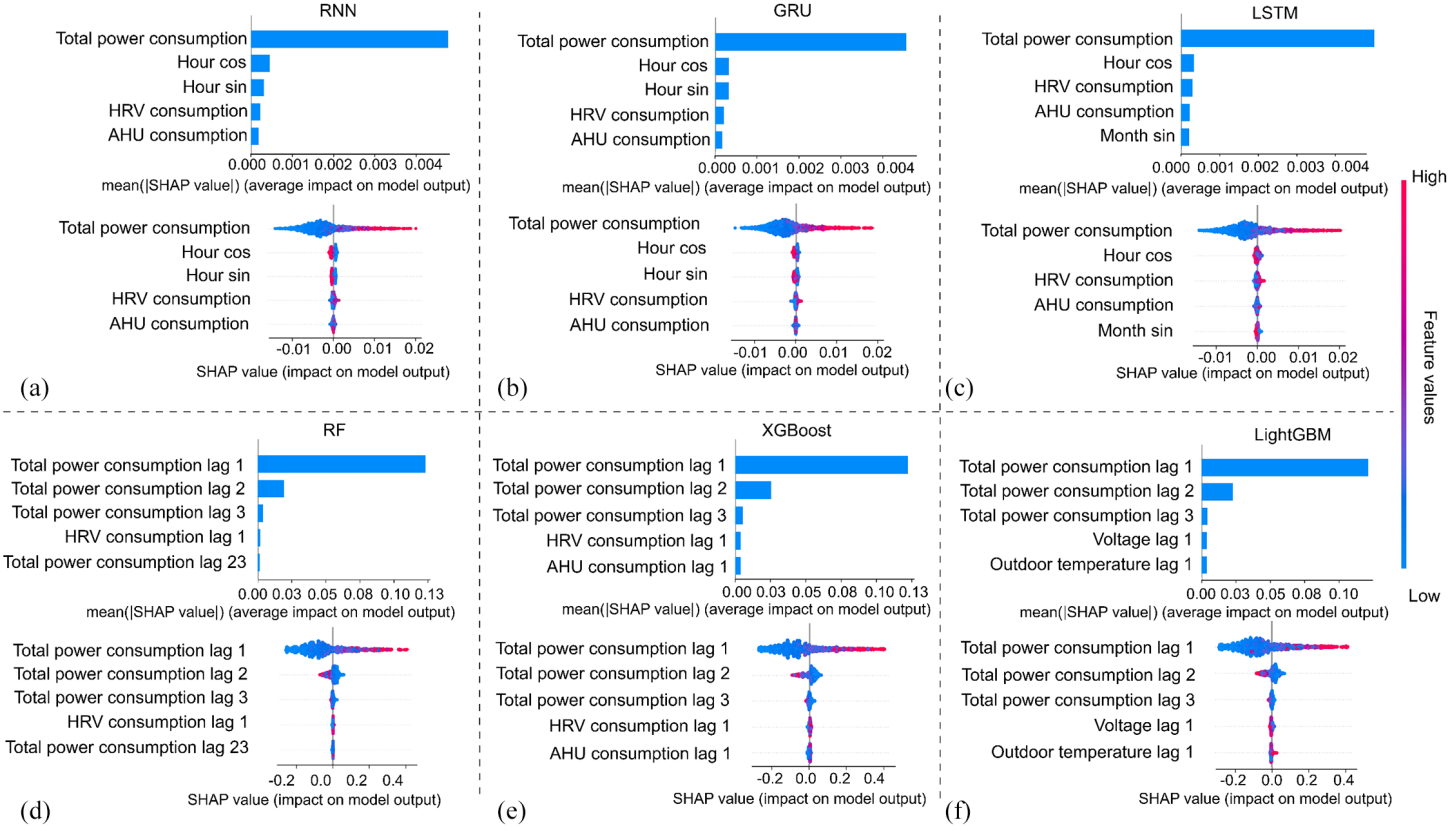
SHAP bar and summary plot for (a) RNN, (b) GRU, (c) LSTM, (d) RF, (e) XGBoost, and (f) LighGBM models.

Figure 6 displays a comprehensive analysis of feature contributions using SHAP values for both tree-based and DL models. The SHAP bar chart (upper chart) visualizes the global importance of the top features by calculating and summing the absolute values across all samples, which helps us understand which features consistently have the most influence on the model’s predictions. Each dot in the SHAP summary plot (lower chart) represents a SHAP value of a single sample from the dataset, with the color gradient ranging from blue to red, indicating low to high feature values. The features are arranged vertically in order of importance, with more important features at the top. The horizontal spread of dots reflects the distribution of SHAP values for each feature, with higher absolute values indicating a more significant influence on the model’s output. It is worth mentioning that all of the features shown in Figure 6 are from the previous 24-time steps. Taking the feature *Total power consumption* of the RNN model as an example, it is observed that higher values of total power consumption significantly affect the prediction. An increasing trend in previous power consumption indicates a higher likelihood of increased consumption in the next hour. Previous power consumption emerges as the dominant feature for forecasting future power usage across DL and tree-based models. In particular, the tree-based models can show that the most recent power consumption data (i.e. power consumption at lags 1, 2, and 3) is the majority input determining the model to make the prediction when compared to the furthest previous power consumption in the summary plot. The mean SHAP value of the top two features can be found in Table 6.

**Table 6.** The mean SHAP values of the top two important features.

Top two important features	Deep learning model		
	RNN	GRU	LSTM
Total power consumption	0.00429	0.00432	0.00423
Hour cosine	0.000351	0.000498	0.000278
Top two important features	Tree-based model		
	RF	XGBoost	LightGBM
Total power consumption lag 1	0.120	0.122	0.118
Total power consumption lag 2	0.018	0.020	0.022

#### Local interpretation

**Figure 7.**
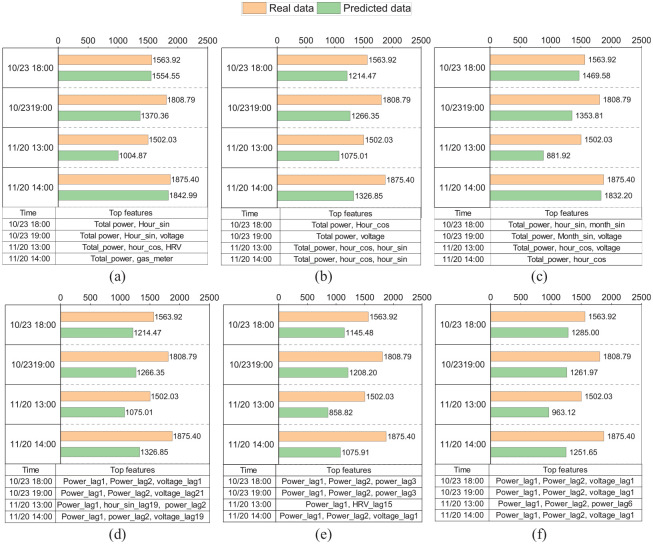
Local explanation for (a) RNN, (b) GRU, (c) LSTM, (d) RF, (e) XGBoost, and (f) LighGBM models.

While global interpretation provides an overall understanding of the model’s performance, it may overlook the specifics of individual predictions. Local explanations, like those given by SHAP values, can help us understand how different features affect the model’s output for each observation, especially when demand is high. Figure 7 depicts a detailed local explanation for six different models. Each subplot in Figure 7 illustrates the prediction accuracy for a specific observation and includes a corresponding table shown in Figure 7 that ranks the features based on their importance at those particular moments. The local interpretation analysis uses four observations as examples, highlighting the differences in feature importance among models.

Different models have different important features for a specific prediction. For example, at the time step of 11/20 at 13:00, the RNN model uses previous power consumption, “hour_cos,” and “HRV” data for its prediction, whereas the LSTM model employs the previous power consumption, “hour_sin,” and “hour_cos.” There is a noticeable difference in the sets of top features identified by DL models and those by tree-based models. While the former relies on previous power consumption and time index features, the latter relies on lags in power consumption and voltage. This difference can be crucial in understanding how different models predict and offer insights into model selection based on feature availability and significance. Notably, all DL models consistently identify total power consumption and time-related features like “hour_sin” or “hour_cos” as critical for predicting the subsequent time step. This consistency suggests that these features are fundamental predictors of total power demand, regardless of the DL model used. On the other hand, tree-based models provide greater transparency by indicating which prior power consumption data points are influential in the decision-making process.

While visualizations of global and local feature contributions highlight which features significantly influence predictions, they do not reveal how neural networks work inside. Understanding feature importance is crucial, but it is only one aspect of the interpretative process. Interpreting the model’s structure and how the layers and activation functions contribute to decision-making is equally essential. Although ensemble tree models have more complex structures than simple decision tree, they are generally more interpretable than DL models. DL models may outperform in some applications, but the mechanisms driving their effectiveness often lack transparency. The interpretability of a DL model’s structure, such as layer configuration and activation function selection, remains challenging.

#### Model structure interpretation

**Figure 8.**
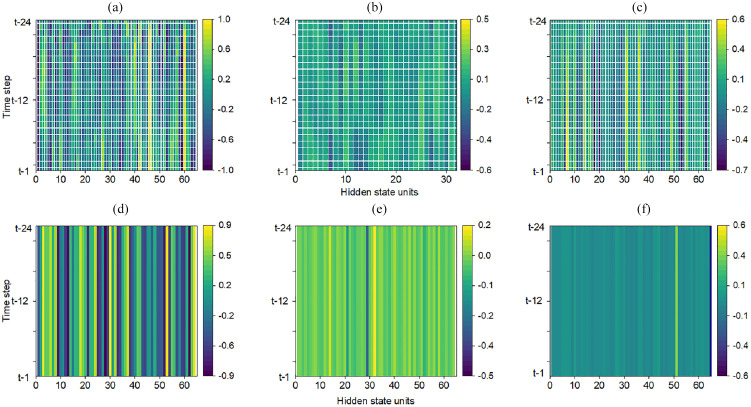
Hidden state visualization for deep learning models: (a) Hidden states heatmap of the first layer of the RNN model, (b) Hidden states heatmap of the first layer of the GRU model, (c) Hidden states heatmap of the first layer of the LSTM model, (d) Hidden states heatmap of the second layer of the RNN model, (e) Hidden states heatmap of the second layer of the GRU model and (f) Hidden states heatmap of the second layer of the LSTM model.

Another method for model explanation is to interpret the structure of the model. The most common approach for ensemble-based tree models is to visualize the decision trees to trace the decision-making process from a high-level perspective. Each node’s decision criteria (splits) are visible, and the path through the tree provides a clear explanation for each prediction. For the deep learning models, we could visualize the hidden state values (often as a heatmap) to interpret the model’s internal representations. The hidden state heatmap can provide insight into the “black-box” of DL models and be helpful in model debugging. Figure 8 shows heatmaps of the hidden states of the first and second layers for three DL models. These visualizations display the hidden state activations across time steps ( Y -axis), representing an hourly data point in the 24-h input sequence used for prediction, and hidden units ( X -axis), with color intensity reflecting the magnitude of activation. Bright areas indicate a strong influence on the model’s output for specific features at specific times, whereas dimmer areas indicate less influence. Inconsistent activity in specific regions (extreme darkness or brightness) indicates that specific features are not influencing the model’s decision-making process. Each unit maintains a hidden state across the input sequence in the first layer. These hidden states can capture different information from the input sequence, allowing the network to remember certain aspects of the input data over time. Obviously, the importance of the hidden states changes over time among these models, meaning that they process the sequences differently since different inputs in the sequence can carry varying amounts of information in different models. The 60th hidden state unit is crucial for the RNN model since the hidden values are very high across the time step, but it is not important for GRU and LSTM models. Furthermore, if a certain column is consistently bright or dark, it is evident that the model is paying more attention to (bright) or ignoring (dark) specific features over time. For example, the 60th hidden unit in the RNN model has a higher overall value, indicating that this hidden unit has a significant effect on the output. It is worth noting that the same hidden units have different influences at each layer. For instance, the 60th hidden state of the RNN model significantly influences the first layer but is less important on the second layer, implying that the layers’ roles are different. The first layer can learn the patterns directly from the input data, while the second layer condenses the information into a single vector and captures only the most relevant information for prediction. Therefore, the importance of a particular unit may change as the data moves through the layers, leading to a uniform color of the hidden state heatmap at the second layer.

**Figure 9.**
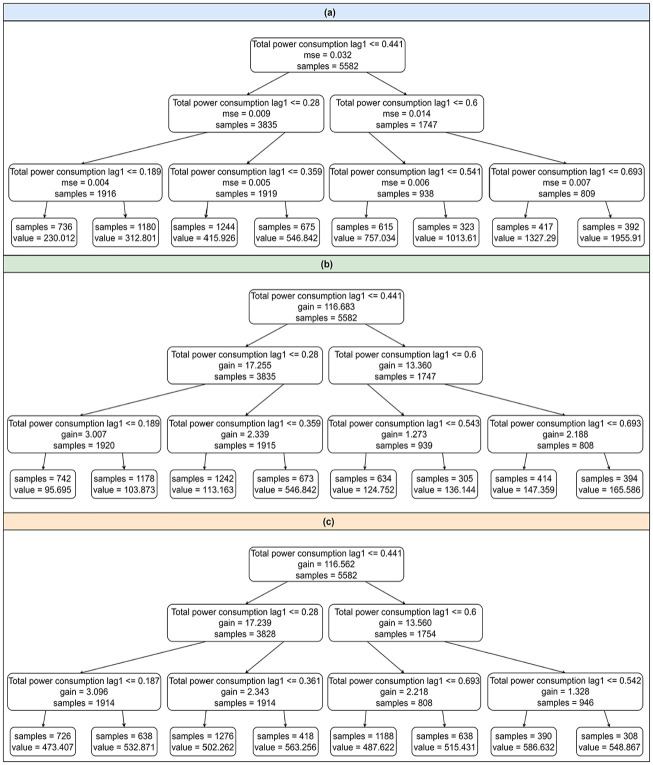
Tree-based model structure. (a) RF tree structure (b) XGBoost tree structure(c) LightGBM tree structure.

Even though we can see a bright color in the heatmap of a hidden unit indicating that some specific features are important to the output, the exact features cannot be discovered because the model’s decision-making process is unknown. In contrast, as shown in Figure 9, the tree structures of tree-based models provide clear decision rules derived directly from the data. It is important to mention that the tree structures are selected partially for demonstration purposes to show the models’ decision-making processes because it is impractical to display the complete structures at once. Clearly, all models use “Total power consumption lag1” at the root and nodes, indicating that this feature is a strong predictor across models. Each node represents a straightforward decision rule. For instance, “Total power consumption lag1 ≤0.28” indicates that the model splits the data into two paths based on whether the total power consumption lag1 is less than or equal to 0.28. Even though these three models have a similar split condition at nodes, the predicted values at leaves differ due to differences in the algorithms’ mechanisms (e.g. bagging approach for RF and boosting approach for XGBoost and LightGBM). The splitting decision is based on the values of the features, but the predictions depend on the algorithm’s learning process.

### Computational efficiency

**Figure 10.**
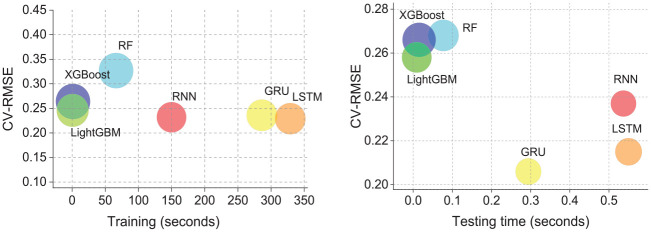
Training (left) and testing (right) time for tree-based and DL models. The circle size reflects the standard deviation of the error.

A model’s efficiency is measured not only by its prediction accuracy but also by the resources it consumes during training and testing. Figure 10 compares various models’ computational time (recorded in seconds) to their performance metrics. DL models have a lower CV-RMSE, indicating a more accurate prediction during the training and testing phases. However, they take significantly longer to train than tree-based models. For instance, as shown in Table 7, the first number is the computational time in seconds. When setting the computation time of LightGBM—the most efficient tree-based model with the lowest error—as a baseline (“1”), the values in brackets indicate the multiple relatives to the baseline. Training times for DL models such as RNN, GRU, and LSTM are 180 to 369 times longer than the baseline. Similarly, their testing times range from 33 to 61 times longer. Furthermore, the reliability of DL is slightly better than that of the tree-based model (the circle size indicates more consistent prediction and reliable performance, a smaller circle denotes more consistent and reliable performance), meaning that tree-based models have similar reliability to DL models.

**Table 7.** Computation time of the models for case study 1.

Model	Algorithm	Training	Testing
Deep learning	RNN	149.99 (180.7)	0.537 (35.8)
	GRU	286.243 (344.8)	0.294 (19.6)
	LSTM	329.003 (369.3)	0.550 (36.7)
Tree-based	RF	66.353 (79.9)	0.077 (5.13)
	XGBoost	1.353 (1.6)	0.09 (6)
	LightGBM	1.830 (1)	0.015 (1)

In Appendix D: Computational efficiency for case study 2, the RF model is set as the baseline due to its high computational efficiency. The DL models, in comparison, require more time for training and testing. The time taken by DL models ranges from 97 to 287 times and 18 to 39 times longer than that of the RF model on training and testing datasets, respectively.

While this study evaluates the effectiveness of the proposed models in predicting power consumption, it is important to acknowledge the limitations. First, there is a limitation in generalizing the results to other building types. The dataset used for training and testing the models is specific to certain residential building types and does not fully represent the diversity of other residential building types (e.g. multi-family apartments) or non-residential buildings, such as commercial or industrial buildings. Second, the dataset used in this study is limited to a specific geographic location and cold climate conditions, which may affect the models’ performance when applied to regions with different climates.

## Conclusion and future work

Achieving a balance between accuracy, interpretability, and computational efficiency is critical for power demand prediction models in building energy management. With the growth of expansive datasets and powerful computational capabilities, ML algorithms have become instrumental in forecasting building power demand. Despite achieving remarkable accuracy, the intricate structure of many advanced models poses challenges in terms of interpretability and computational demand, evaluating the models’ performance solely based on prediction accuracy is insufficient. This study used data from two residential units as the case study to train and test three DL models and three tree-based models, which were judged on how accurate they were, how easy they were to understand, and how quickly they could do their work. The following are the main conclusions. The following are the main conclusions:

1. Due to the nature of the data, there was no consensus on a single “best” model, as performance varied across case studies. This indicates that the models do not consistently outperform each other in all scenarios. Although DL models marginally surpass tree-based models in overall accuracy, the best-performing prediction model varies under different power demand levels. In scenarios characterized by lower power levels and simpler usage patterns, such as sinusoidal consumption patterns, tree-based models can be equally accurate as DL models.
2. From the perspective of model interpretation. Both DL and tree-based models consistently identify previous power consumption as a critical predictor. Nevertheless, tree-based models provide greater clarity at specific observations, revealing the impact of specific lagged power consumptions with clarity. The inherent transparency of tree-based models benefits energy managers and operators by allowing them to make optimized decisions to improve building energy efficiency.
3. Due to the fact that DL models have more parameters that require more computational resources, training times for DL models were found to be 180 to 369 times longer and 97 to 287 times longer than those for tree-based models in case studies 1 and 2, respectively. When large-scale energy modeling or frequent model updating is required, this factor is critical.

In future studies, firstly, given the difficulty of forecasting peak demand, probabilistic methodologies may be considered as alternatives to point predictions. These approaches provide a probabilistic range of outcomes as well as a way to quantify uncertainty. Second, causal inference techniques can illustrate cause-and-effect relationships between variables, which helps better understand how the prediction changes if a feature is intervened. Additionally, treating holiday data as a feature could allow the models to learn specific patterns associated with holidays, such as changes in occupant behavior due to festive activities. Considering these conclusions, tree-based models should be given more attention in predicting building energy consumption. It is noteworthy that they can achieve a balance between accuracy, interpretability, and computational efficiency. This paper provides a valuable reference for using machine learning algorithms in building energy demand forecasting and offers multiple perspectives on model selection trade-offs.

## Figures and Tables

**Figure A1.**
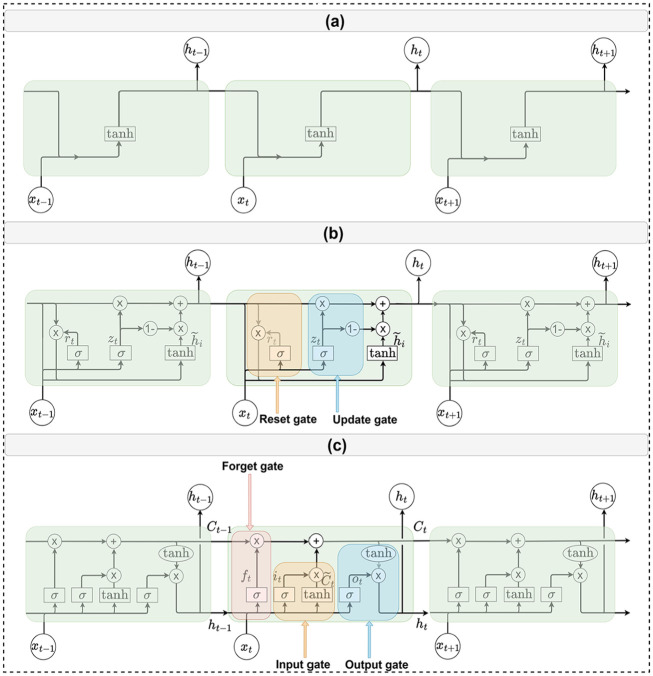
The model architecture of RNN, GRU, and LSTM: (a) RNN model, (b) GRU model, and (c) LSTM model.

**Figure A2.**
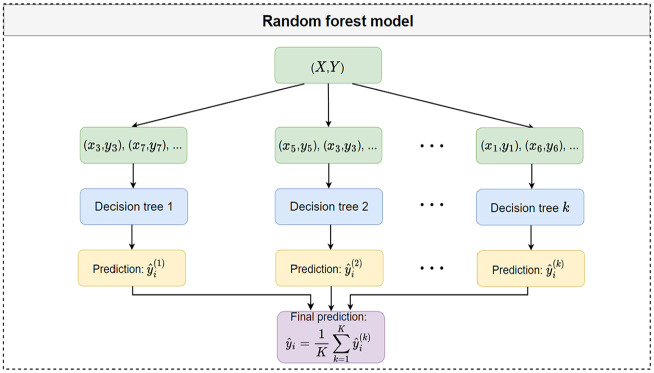
The tree structure of RF.

**Figure A3.**
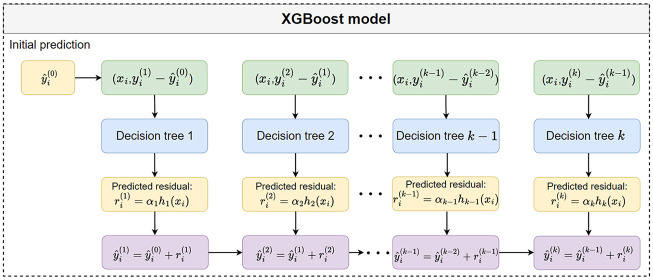
The tree structure of XGBoost.

## Appendices

### Appendix A: Details of the ML models’ principles

#### Recurrent neural network model

An RNN is characterized by three sets of weights: $W_{x}$ , $W_{h}$ , $W_{y}$ are the weights of the current input, hidden state, and output, respectively, and the RNN’s architecture is shown in Figure A1(a). As shown in equation (A1), the RNN updates the hidden state $h_{t}$ at each time step t based on the current input $x_{t}$ and the previous hidden state $h_{t-1}$ .

(A1)
$$h_{t}=\tanh(W_{x}\cdot x_{t}+W_{h}\cdot h_{t-1}+b_{h})$$

After hidden state computation, the RNN produces an output $y_{t}$ using the current hidden state $h_{t}$ , as defined by equation (A2).

(A2)
$$y_{t}=\sigma (W_{y}\cdot h_{t}+b_{y})$$

The bias terms $b_{h}$ and $b_{y}$ are added to the model to give it more freedom and allow it to better fit the data.

#### Gated recurrent unit model

Figure A1(b) depicts the structure and calculation process of GRU. Each GRU unit contains weight matrices $W_{z}$ , $W_{r}$ , and $W_{h}$ , which correspond to the update gate, reset gate, and candidate hidden state, respectively, and $b_{z}$ , $b_{r}$ , and $b_{h}$ are the bias vectors. The process of GRU begins with determining which hidden state in the update gate should be updated with the new hidden state candidate, as described by equation (A3).

(A3)
$$z_{t}=\sigma (W_{z}\cdot [h_{t-1},x_{t}]+b_{z})$$

where, σ represents the sigmoid function, $h_{t}-1$ represents the previous hidden state, and $x_{t}$ represents the current input. The reset gate then decides how much of the previous hidden state should be discarded, as shown in equation (A4).

(A4)
$$r_{t}=\sigma (W_{r}\cdot [h_{t-1},x_{t}]+b_{r})$$

The candidate for the new hidden state is then computed using equation (A5).

(A5)
$$\tilde{h_{t}}=\tanh\;(W_{h}\cdot \left[r_{t}\times h_{t-1},x_{t}\right]+b_{h})$$

where, $r_{t}\times h_{t-1}$ is an element-wise multiplication indicating the effect of the reset gate on the previous hidden state.

Finally, the actual hidden state at time t , $h_{t}$ is a combination of the previous hidden state $h_{t-1}$ and the new hidden state candidate $\tilde{h_{t}}$ , weighted by the update gate $z_{t}$ , as defined in equation (A6).

(A6)
$$h_{t}=\left(1-z_{t}\right)\times h_{t-1}+z_{t}\times \tilde{h_{t}}$$

The GRU iteratively processes each element of the input sequence, passing the hidden state forward through time, and allowing information to be carried across different time steps.

#### Long short-term memory model

Figure A1(c) depicts the structure and calculation process of LSTM. The LSTM starts with determining what information the cell state can keep or forget in the forget gate, and the initial values of the weight matrices (i.e. $W_{f},W_{i},W_{C},W_{o}$ ) and bias (i.e. $b_{f},b_{i},b_{C},b_{o}$ ) are updated using the gradient descent method. The previous hidden state $h_{t-1}$ and the current input $x_{t}$ are passed through a sigmoid function, as shown in equation (A7).

(A7)
$$f_{t}=\sigma (W_{f}\cdot \left[h_{t-1},x_{t}\right]+b_{f})$$

where the $f_{t}$ is the output with a number between 0 and 1, indicating whether the information is kept (close to 1) or forgotten (close to 0). The input gate provides new information to the cell state in two steps: First, the previous hidden state and the current input are sent to a sigmoid function to decide what relevant information needs to be updated using equation (A8). Second, the previous hidden state and the current input are sent to the tanh function to generate new candidates $\tilde{C_{t}}$ using equation (A9).

(A8)
$$i_{t}=\sigma (W_{i}\cdot \left[h_{t-1},x_{t}\right]+b_{i})$$

(A9)
$$\tilde{C_{t}}=\tanh(W_{C}\cdot \left[h_{t-1},x_{t}\right]+b_{C})$$

The next step is to update the old cell state $\tilde{C_{t}}$ to a new cell state $C_{t}$ . It multiplies the old state by $f_{t}$ and adds the $i_{t}*\tilde{C_{t}}$ , equation (A10).

(A10)
$$C_{t}=f_{t}*C_{t-1}+i_{t}*\tilde{C_{t}}$$

Lastly, the output gate and the updated cell state determine the current hidden $h_{t}$ , as shown in equation (A11) and equation (A12), to predict the building power demand at t+1 . The cell state and hidden state are passed to the next time step, allowing information to persist.

(A11)
$$o_{t}=\sigma (W_{o}\cdot \left[h_{t-1},x_{t}\right]+b_{o})$$

(A12)
$$h_{t}=o_{t}*\tanh(C_{t})$$

#### Random forest model

As shown in Figure A2. The process of training RF has two techniques: bootstrapping and bagging. Bootstrapping is a resampling technique that generates multiple subsets from the original dataset through random sampling with replacement. After that, each DT is trained on a different bootstrapped dataset, which enhances the robustness of the RF model and reduces the risk of overfitting. Upon training the individual trees on the bootstrapped samples, RF aggregates their predictions, and this aggregation step reduces the variance of the predictions and makes the final model more stable and accurate.

- The detailed process of the RF algorithm is as follows:
- **Initialization**: The RF model is initialized with a dataset containing the feature set X and the target variable Y .
- **Bootstrap sampling**: Observations are randomly sampled with replacement from the original dataset for each decision tree, denoted as tree k , where k ranges from 1 to K (the total number of the trees in the forest). This method ensures that each tree is trained on a slightly different set of data, adding to the model’s diversity.
- **Tree development**: Each tree is built independently using the bootstrap sample. Traditional DT criteria are employed to determine the best splits during this development phase.
- **Final prediction**: Once all trees have been trained, the model takes the average of all the predictions to obtain the final prediction, which is expected to be more accurate and stable than a single tree’s prediction.

#### Extreme gradient boosting model

As shown in Figure A3. The prediction ${\hat{y}}_{i}$ for the ith observation after K regression trees for a set of n observations is defined as follows:

(A13)
$${{\hat{y}}_{i}}^{\left(k\right)}\;=\;\sum_{k=1}^{K}f_{k}\left(x_{i}\right)={{\hat{y}}_{i}}^{\left(k-1\right)}+\alpha_{k}\cdot h_{k}(x_{i})$$

where K represents the number of regression trees, $f_{k}\left(x_{i}\right)$ is the prediction function of the $k^{\mathrm{th}}$ decision tree for input $x_{i}$ , and ${{\hat{y}}_{i}}^{\left(k\right)}$ is the cumulative predicted value at the $k^{\mathrm{th}}$ decision tree. The term $h_{k}\left(x_{i}\right)$ is the function trained to predict the residual $r_{k-1}$ , and $\alpha_{k}$ is the learning rate. The objective function of XGBoost is to assess how well the model predicts the target value. The objective function in XGBoost includes two parts: a loss function and a regularization term. It is defined as follows:

(A14)
$$\mathrm{Objective}\mathrm{function}=\sum_{i=1}^{n}L\left(y_{i},{\hat{y}}_{i}\right)+\sum_{k=1}^{K}\Omega (f_{k})$$

where $\sum_{i=1}^{n}L\left(y_{i},{\hat{y}}_{i}\right)$ is used to measure the difference between the predicted value and the actual value of the loss function. $\sum_{k=1}^{K}\Omega (f_{k})$ is the regularization term to prevent overfitting. The calculation of the regularization term is shown in equation (A15).

(A15)
$$\Omega \left(f_{k}\right)=\gamma T+\frac{1}{2}\lambda {\left\|\omega \right\|}^{2}$$

where T denotes the number of terminal nodes or leaves in a tree, while γ and λ are hyperparameters that penalize model complexity, avoiding overfitting.

The detailed processes of the XGBoost algorithm are as follows:

- **Initial prediction**: Before building any trees, start with the initial prediction for all observations before any trees are constructed, denoted as ${{\hat{y}}_{i}}^{\left(0\right)}$ . It is usually the mean or median of the target variable.
- **First tree**: The first DT attempts to predict the residual based on the initial prediction. This tree’s prediction is combined with the initial prediction to produce an updated prediction, ${{\hat{y}}_{i}}^{\left(1\right)}$ .
- **Second tree**: Similarly, the second DT focuses on predicting the residuals from the first tree’s predictions. It attempts to correct the errors made by the first tree and ${{\hat{y}}_{i}}^{\left(2\right)}$ denotes the updated prediction after the second tree.
- $k^{\mathrm{th}}$ **tree**: This process is repeated for the $k^{\mathrm{th}}$ tree. The prediction at $k^{\mathrm{th}}$ tree is the sum of the initial prediction and the contribution from all previous trees.

## Appendix B: Overall prediction accuracy comparison for case study 2

**Table B1.** Evaluation of the accuracy metrics for the prediction models for case study 2.

Metrics	Validation					
	DL-based Models			Tree-based models		
	RNN	GRU	LSTM	RF	XGBoost	LightGBM
MAE	103.21	78.47	79.93	77.73	101.07	78.75
MAPE	8.09%	8.89%	8.18%	8.58%	11.47%	8.86%
RMSE	103.21	112.39	123.43	117.79	147.00	117.10
NMBE	3.42%	−4.01%	−5.26%	1.27%	−0.90%	0.55%
CV-RMSE	12.5%	13.62%	13.39%	14.03%	17.52%	13.96%
Metrics	Testing					
	RNN	GRU	LSTM	RF	XGBoost	LightGBM
MAE	207.97	154.17	151.34	184.38	240.39	175.53
MAPE	10.90%	8.05%	8.186%	9.39%	12.10%	8.83%
RMSE	268.41	207.30	212.17	260.06	347.84	262.03
NMBE	9.18%	3.40%	−1.01%	6.25%	7.21%	5.64%
CV-RMSE	14.30%	11.07%	11.34%	13.70%	18.32%	13.80%

## Appendix C: Prediction accuracy at different power level for case study 2

**Table C1.** Prediction performance at different power levels for case study 2.

Powerlevel (W)	Metrics	DL-based models			Tree-based models		
		RNN	GRU	LSTM	RF	XGBoost	LightGBM
<500	MAE	72.29	87.69	71.62	125.52	216.48	162.00
	MAPE	19.32%	23.79%	19.39%	32.75%	56.66%	43.75%
	RMSE	80.05	102.15	85.01	139.33	236.81	180.26
	NMBE	−15.19%	−21.71%	−17.86%	−30.35%	−54.64%	−40.88%
	CV-RMSE	20.20%	25.78%	21.45%	35.16%	59.77%	45.50%
500–1000	MAE	100.39	80.43	94.74	117.44	169.86	94.75
	MAPE	12.58%	10.51%	12.10%	15.20%	22.25%	12.52%
	RMSE	121.86	109.12	123.87	148.96	209.68	129.29
	NMBE	6.88%	−2.25%	−4.21%	−5.60%	−15.14%	−4.35%
	CV-RMSE	15.11%	13.53%	15.36%	18.48%	26.01%	16.04%
1000–1500	MAE	147.47	92.80	111.07	96.76	115.95	101.12
	MAPE	11.36%	7.16%	8.56%	7.48%	9.01%	7.79%
	RMSE	174.43	124.59	150.52	125.64	148.74	132.84
	NMBE	9.27%	−0.62%	−3.38%	1.12%	−2.20%	2.29%
	CV-RMSE	13.37%	9.55%	11.53%	9.63%	11.40%	10.18%
1500–2000	MAE	188.39	133.62	138.30	153.30	153.02	134.43
	MAPE	10.75%	7.61%	7.95%	8.76%	8.68%	7.72%
	RMSE	226.60	168.56	182.47	187.54	193.51	171.53
	NMBE	9.08%	2.21%	−1.33%	5.66%	4.79%	3.65%
	CV-RMSE	12.99%	9.66%	10.46%	10.75%	11.09%	9.83%
>2000	MAE	227.64	221.07	196.21	277.28	402.95	267.43
	MAPE	10.57%	8.54%	7.57%	10.33%	15.07%	9.66%
	RMSE	351.68	277.4	270.51	366.51	513.62	374.77
	NMBE	9.34%	5.69%	0.13%	8.83%	12.67%	8.31%
	CV-RMSE	13.75%	10.84%	10.57%	14.33%	20.08%	14.65%

## Appendix D: Computational efficiency for case study 2

**Table D1.** Computation time of the models for case study 2.

Model	Algorithm	Training	Testing
Deep learning	RNN	154.67 (97.76)	0.258 (18.42)
	GRU	343.276 (216.98)	0.406 (29)
	LSTM	454.57 (287.33)	0.555 (39.64)
Tree-based	RF	9.153 (5.78)	0.128 (9.142)
	XGBoost	1.582 (1)	0.014 (1)
	LightGBM	2.056 (1.29)	0.020 (1.42)
